# Physiological and biological characterization of smokers with and without COPD

**DOI:** 10.12688/f1000research.11698.2

**Published:** 2018-03-07

**Authors:** Nveed Chaudhary, Karsta Luettich, Michael J. Peck, Elena Pierri, Loyse Felber-Medlin, Gregory Vuillaume, Patrice Leroy, Julia Hoeng, Manuel C. Peitsch

**Affiliations:** 1 Philip Morris Products SA, Philip Morris International R&D, Quai Jeanrenaud 5, 2000 Neuchâtel, Switzerland

**Keywords:** COPD, lung function, gas transfer, impulse oscillometry, lung sound analysis, biomarker

## Abstract

Chronic obstructive pulmonary disease (COPD) is a common inflammatory airway disease predominantly associated with cigarette smoking, and its incidence is increasing worldwide. According to the Global Initiative for Obstructive Lung Disease (GOLD) guidelines, spirometry is used to diagnose the disease. However, owing to its complexity, spirometry alone may not account for the multitude of COPD phenotypes or the early, asymptomatic lung damage seen in younger smokers. In addition, suitable biomarkers enabling early diagnosis, guiding treatment and estimating prognosis are still scarce, although large scale ‘omics analyses have added to the spectrum of potential biomarkers that could be used for these purposes.

The aim of the current study was to comprehensively profile patients with mild-to-moderate COPD and compare the profiles to i) a group of currently smoking asymptomatic subjects, ii) a group of healthy former smokers, and iii) a group of healthy subjects that had never smoked. The assessment was conducted at the molecular level using proteomics, transcriptomics, and lipidomics and complemented by a series of measurements of traditional and emerging indicators of lung health (ClinicalTrials.gov identifier: NCT01780298). In this data note, we provide a comprehensive description of the study population’s physiological characteristics including full lung function, lung appearance on chest computed tomography, impulse oscillometry, and exercise tolerance and quality of life (QoL) measures.

## Introduction

Chronic obstructive pulmonary disease (COPD) is a respiratory disease characterized by progressive airflow limitation and is associated with an abnormal inflammatory response of the lung to noxious particles and gases. Globally, airflow obstruction can be seen in approximately 25% of adults aged 40 and over ^1^, and the prevalence of COPD is on the rise worldwide, leading to predictions of COPD becoming the third leading cause of death by 2030 ^2^.

The clinical assessment of a patient with suspected obstructive lung disease relies on symptoms such as shortness of breath and persistent cough, medical history, history of risk factors (e.g. cigarette smoking) and spirometry. The latter, based on the latest recommendations of the Global Initiative for Obstructive Lung Disease (GOLD) guidelines ( www.goldcopd.org), is required for confirming a COPD diagnosis in case of a post-bronchodilator ratio of forced expiratory volume in 1 second over forced vital capacity (FEV _1_/FVC) of less than 0.7 or 70%. The efforts of GOLD to simplify the diagnosis of COPD to a single repeatable test that uses inexpensive equipment in the physician’s office have proved critical and invaluable in the day-to-day diagnosis and management of the disease. However, it has become clear that COPD is a very complex, heterogeneous disease consisting of a multitude of different phenotypes and syndromes, even among subjects with a similar degree of airflow limitation, and with highly variable rates of progression ^3^. Spirometry alone may also not be sufficiently sensitive to account for early lung damage that remains asymptomatic, particularly in the younger smoker ^4^. Moreover, airflow obstruction does not correlate well with clinical outcomes such as frequency of exacerbations and mortality ^5^. It is not surprising then that pharmacological interventions are rather modestly successful and long-term, positive patient-centric outcomes are infrequently achieved ^6^. Similarly, and although our mechanistic understanding of COPD pathophysiology is ever-increasing, the identification of suitable biomarkers for the diagnosis, treatment and prognosis of the disease is still lagging behind compared to other areas of clinical research ^7^. However, these gaps in our knowledge have long been recognized and were recently highlighted as key areas for further research, together with recommendations for how to address them ^6^. In addition, there is a clear call for the application of novel, sophisticated approaches to precision medicine to aid in answering some of these questions ^7^.

The study that we conducted was designed with at least some of these aspects in mind, aimed at the identification of a biomarker or a panel of biomarkers for the differentiation of subjects with mild to moderate COPD, current smokers, former smokers and never-smokers, using gene and protein expression analyses in various biological samples together with the assessment of traditional and emerging indicators of lung health. In this data note, we provide a comprehensive description of the study population and their physiological characteristics, including full lung function, lung appearance on chest computed tomography, impulse oscillometry, exercise tolerance and different quality of life measures. We also introduce lung sound analysis (stethographics) as a potential approach to identify subjects with subclinical disease that would be missed by considering spirometry outcomes on their own.

The results of proteomics and lipidomics analyses were published here ^8^ and here ^9^, and transcriptomics results are currently in press ^10^.

## Materials and methods

This study used a parallel-group, case-controlled study design to assess a number of established and potentially novel biomarkers in smokers with COPD and in various control populations (never-smokers, former smokers, and asymptomatic current smokers). In this data note, we provide data of physiological measurements and quality of life (QoL) for the 240 subjects who completed the study. Following approval from a UK National Health Service (NHS) Ethics Committee (The Black Country Ethics Committee), the study was conducted as a single center study in strict compliance with Good Clinical Practice (GCP) guidelines.

 The study has been registered on ClinicalTrials.gov with identifier NCT01780298 (trial registration date: 21 January 2013).

Potentially suitable subjects were identified for inclusion into the study via the study center’s database and by media advertising. Subjects of both genders (41–70 years old) were enrolled in this study, starting with the COPD study group. Never-smokers, former and current smokers were then enrolled aiming to match the subjects with COPD by age (±5 years), ethnicity, and gender. The smoking history of all smoking subjects was at least 10 pack-years. Former smokers had to have quit smoking at least one year prior to the study. Subjects that discontinued participation (for medical or personal reasons) were replaced. 

Additional information regarding the recruitment process, including inclusion and exclusion criteria for this study and information regarding withdrawal or removal of subjects are provided in the Supplementary Material (sections S1 to S3).

The study comprised a maximum of 5 out-patient visits to the study center. The initial visit served to inform the potential participant about the study and the potential risks as well as to obtain informed consent. Once informed consent was obtained, subject screening including recording of demographic data, vital signs, weight and height, determination of medical and surgical history, physical examination, and measurement of forced expiratory volume in 1 second (FEV _1_) and forced vital capacity (FVC) took place. Further, a 12-lead electrocardiogram (ECG), laboratory assessments and urinalysis were performed. Females of childbearing potential underwent a serum pregnancy test, and all subjects underwent alcohol breath test, plasma cotinine test and a drugs of abuse test. Smokers were provided with information about smoking cessation. Finally, subjects were asked to provide ≥0.1 g of sputum sample. For subjects identified as having COPD based on screening assessments and who had their diagnosis documented by the investigator, the subject’s general practitioner was notified in writing of the assessment results and clinical conclusions. This first visit was followed by visits 1a or 1b to give subjects another opportunity to produce an adequate sputum sample and to reassess non-smokers previously deemed ineligible based on sputum neutrophil counts, respectively.

At visit 2 and prior to all other procedures, eligibility was reassessed and the questionnaires were completed. Vital signs and differential fractional exhaled nitric oxide (FENO) were recorded; full lung function including transfer factor (carbon monoxide diffusing capacity [T _L_CO]) and exhaled breath temperature (EBT) were measured, and subjects were asked to undergo impulse oscillometry (IOS) and computerized multichannel lung sounds analysis (stethographics). In addition, during this visit, induced sputum, blood, and nasal samples were collected for subsequent analysis of inflammatory markers and ‘omics including transcriptomics, proteomics and lipidomics.

During visit 3, subjects underwent a high-resolution computerized tomography (HRCT) lung scan and cardio-pulmonary exercise testing with an electronically braked cycle ergometer. Twelve-lead ECG, vital signs and physical examination were performed before and after the exercise test.

Assessments at visit 4 included measurement of vital signs, differential FENO, full lung function, IOS, stethographics, EBT, nasal sampling, and sputum induction.

A follow-up telephone call approximately 3 to 10 days after visit 4 concluded the study, and a summary letter was sent to their general practitioner by the investigator. Throughout the study, smokers received smoking cessation advice, and any adverse events (AEs) were recorded.

The full schedule of events is provided in the Supplementary Material (section S4).

**Figure 1.**
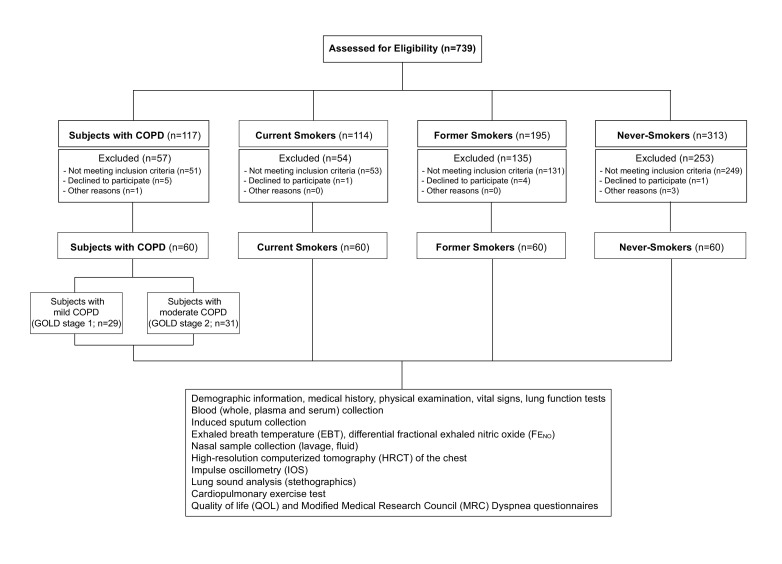
CONSORT flow diagram of the progress through the phases of this study (i.e. enrolment, allocation, visits/measurements, and data analysis).

The summary of the study enrolment and recruitment success, as well as a summary of the samples collected from each of the 240 subjects and the physiological and clinical measurements that were taken are depicted in Figure 1, following the 2010 Consolidated Standards of Reporting Trials (CONSORT) guidelines ^11^.

### Full lung function

Spirometry was performed pre- and post-bronchodilator administration in all subjects at visit 1 to meet ATS/ERS criteria ^12^. Predicted values for FEV _1_ and FVC were calculated according to the formula of the European Coal and Steel Community (ECSC) ^13^.

The multiple-breath inert gas washout (helium dilution) technique measuring the functional residual capacity (FRC) of the lungs was employed to determine lung volume. The subjects were asked to breathe normally into a closed circuit spirometer connected to a re-breathing bag filled with 9% helium and 21% oxygen. After tidal breathing, subjects performed an expiratory reserve volume (ERV) maneuver over the next 30 seconds, and a trend of the helium wash-in curve and the helium value was obtained.

For gas transfer measurements, after a few tidal breaths, each subject was asked to take a breath in and then exhale as far as possible, continuing until they felt that the lungs were completely empty. This maximal exhalation was considered the residual volume (RV). The subject was then asked to inhale as far as possible which estimated at least 90% of the subject’s vital capacity (VC) followed by holding their breath for 10 seconds without straining. Finally, the subjects were asked to exhale as far as possible. On completion of the maneuver, the subject was allowed to rest for at least 4 minutes and remain seated before repeating the test at least twice.

### Impulse oscillometry

IOS was performed twice during the study, at visits 2 and 4. The subject was asked to put the mouthpiece between his/her teeth and to keep their lips firmly sealed around the mouthpiece and breathe normally while wearing a nose-clip and sitting upright with his/her head straight or slightly extended. Once the subject’s breathing baseline was established, measurements were recorded up to 90 seconds.

### Computerized multichannel lung sound analysis (Stethographics)

Stethographics analysis was performed at visits 2 and 4, using the 16-channel lung sound analyzer system STG1602 (Stethographics, Boston, MA, USA) following the supplier’s recommendations ^14^. Lung sound data were obtained from a normal deep-breathing protocol (pattern 1; P1), cough recording at FVC, and an intermediate deeper-than-normal breath pattern (pattern 2; P2). Each breath pattern was recorded for a minimum of 30 seconds allowing 3 to 6 breaths to be taken, with measurements completed over 3 to 4 minutes. Measurements were taken in a quiet room to minimize the likelihood of movement artefacts and ambient noise and analyzed using the proprietary stethographics software. Each of the 16 parameters derived from one measurement were evaluated and a score from 0 to 10 was assigned based on the value of the individual parameter. The total standard Acoustic COPD Score (ACOPDS) was calculated as the sum of the individual score for each parameter. The maximum score for each parameter was 10; therefore, the maximum possible ACOPDS for each subject was 160.

### High-resolution computerized tomography

Chest scans were obtained using a 64-slice Discovery VCT scanner (GE Healthcare, Little Chalfont, Buckinghamshire, UK) without contrast medium. Scan duration was less than 5 minutes, including positioning the subject. When the subject was acclimatized in a supine position on the CT scanner table, they were instructed on use of the breath-holding procedure required for the scan. A standard HRCT scan of the chest was acquired at 1.25 mm slices every 10 mm on inspiration. Any deviation from standard CT scan parameters was recorded. CT scans were assessed separately by 2 radiologists who were blinded to the subjects’ details and study group assignments. Each HRCT scan was analyzed within 24 hours, and any abnormalities including lung nodules and extrathoracic abnormalities, were reported. A scoring system was applied to grade the various features visible on the scans ^15– 18^:

1.  Extent of disease: Emphysema was defined as areas of decreased attenuation, usually without discrete walls, and of non-uniform distribution causing permeative destruction of lung parenchyma. The extent of emphysema was estimated visually to the nearest 5%. The mean figure was taken as the extent of emphysema. The following score was used: 0 = absent, 1 = mild, 2 = moderate, 3 = severe, 4 = very severe.
2.  Severity of bronchial dilatation was measured in morphologically normal lung and graded semi-quantitatively by comparison with the homologous pulmonary artery. The scores assigned were: 0 = none; 1 = mild (1.5x to 2.5x diameter of pulmonary artery); 2 = severe (>2.5x diameter of pulmonary artery).
3.  Traction bronchiectasis was defined as bronchial dilatation within areas of reticular pattern and was graded by comparison with the homologous pulmonary artery using the same scoring system as for severity of bronchial dilatation.
4.  A total bronchiectasis score was derived by adding up the severity of bronchial dilatation and traction bronchiectasis scores.
5.  Bronchial wall thickening was graded as follows: 0 = none; 1 = 0.5x; 2 = 0.5-1x; or 3 = >1x the diameter of the adjacent pulmonary artery.
6.  Small airways disease was defined as areas of decreased attenuation associated with a reduction in the number and caliber of pulmonary vessels, but without vascular distortion that is seen in centrilobular emphysema ^17^. The extent of decreased attenuation ascribable to small airways disease was estimated visually to the nearest 5% and scored as follows: 0 = absent, 1 = mild, 2 = moderate, 3 = severe and 4 = very severe.
7.  The total COPD score was derived from the means of the component scores.

### Cardiopulmonary exercise tolerance test

The cardiopulmonary exercise tolerance test following the modified Bruce protocol ^19^ was conducted at visit 3 by a clinical physiologist. All phases of the exercise test were completed if possible and if the subject did not experience clinically significant findings or significant fatigue. Maximal oxygen uptake or VO _2max_ was recorded.

### Questionnaires

All subjects completed three questionnaires during the study: 1) A general questionnaire considering education, lifestyle, smoking history and family history of disease; 2) the SF-36 questionnaire - a multi-purpose, short-form QoL health survey ^20^; and 3) the modified Medical Research Council (MMRC) Dyspnoea Scale, a self-grading scale of the degree of breathlessness during activities. The MMRC Dyspnoea Scale uses a simple grading system to assess a subject’s level of dyspnoea (shortness of breath) ^21^. Subjects were asked to grade the degree of breathlessness during activities by choosing one of the following:

1.  Not troubled by breathlessness except on strenuous exercise.
2.  Short of breath when hurrying or walking up a slight hill.
3.  Walks slower than contemporaries on the level because of breathlessness, or has to stop for breath when walking at own pace.
4.  Stops for breath after about 100 meters or after a few minutes on the level.
5.  Too breathless to leave the house, or breathless when dressing or undressing.

### Modified BODE Index

The modified BODE (mBODE) index was derived from the BMI (B), the degree of airflow obstruction indicated by FEV _1_ % predicted (O), functional dyspnea as measured by the MMRC Dyspnoea Scale (D), and exercise capacity reflected by VO _2max_ (E) ^22, 23^, and scored based on the following matrix ( Table 1 ^24^):

**Table 1.** Scoring matrix for the modified BODE index ^24^.

	0	1	2	3
FEV _1_% Pred	>65	50-64	36-49	<35
VO _2max_	>25	20-25	15-20	<15
MMRC Dyspnoea Scale	0–1	2	3	4
BMI	>21	<21

Demographics List of all unique study subject IDs (USUBJID) and demographic information including sex, age, COPD GOLD stage (if any), cigarette consumption (expressed as pack-years and cigarettes per day), study group (ARMCD), and the matching ID (MATCHID) allowing the pairing of control subjects to their ‘matched’ COPD subject. Click here for additional data file. Copyright: © 2018 Chaudhary N et al. 2018 Data associated with the article are available under the terms of the Creative Commons Zero "No rights reserved" data waiver (CC0 1.0 Public domain dedication).

Vital Signs Table of body mass index, diastolic and systolic blood pressure, heart and respiration rate measures. The data for each measurement (TEST) are presented as value (standard result, STRESN) and unit (standard unit, STRESU) for each subject, and visit number (VISITNUM) at which measurements were obtained. Click here for additional data file. Copyright: © 2018 Chaudhary N et al. 2018 Data associated with the article are available under the terms of the Creative Commons Zero "No rights reserved" data waiver (CC0 1.0 Public domain dedication).

Lung Function Table of lung function data for each subject, including percent predicted values for:
-  Forced expiratory volume in one second (FEV1 %Pred)
-  Forced vital capacity (FVC %Pred)
-  Ratio of FEV1 to maximum vital capacity (FEV 1 % VC MAX)
-  Functional residual volume by helium dilution technique (FRC-He %Pred)
-  Maximum expiratory flow at 25% of expired volume (MEF 25 %Pred)
-  Maximum expiratory flow at 75% of expired volume (MEF 75 %Pred)
-  Diffusion capacity (transfer factor) for carbon monoxide (TLCO %Pred)
The data for each measurement (TEST) are presented as value (standard result, STRESN), together with the visit number (VISITNUM) at which measurements were obtained. Click here for additional data file. Copyright: © 2018 Chaudhary N et al. 2018 Data associated with the article are available under the terms of the Creative Commons Zero "No rights reserved" data waiver (CC0 1.0 Public domain dedication).

Impulse Oscillometry Table of impulse oscillometry data for each subject, including resistance at 5 (R at 5Hz) and 20 Hertz (R at 20Hz), reactance at 5 (X at 5Hz), and Resonant Frequency. The data for each measurement (TEST) are presented as value (standard result, STRESN), together with the visit number (VISITNUM) at which measurements were obtained. Click here for additional data file. Copyright: © 2018 Chaudhary N et al. 2018 Data associated with the article are available under the terms of the Creative Commons Zero "No rights reserved" data waiver (CC0 1.0 Public domain dedication).

Stethographics Stethographics measures for each subject were obtained for 2 breathing patterns, also referred to as normal breathing pattern or P1 (P1 : normal) and deeper breathing or P2 (P2 : deeper). Data presented here include scores for:
-  Expiratory crackle rate for P2 breathing pattern [Expir Crackle Rate(P2 : deeper)]
-  Inspiratory crackle rate for P2 breathing pattern [Insp Crackle Rate(P2 : deeper)]
-  Expiratory wheeze rate for P2 breathing pattern [Expir Wheeze Rate(P2 : deeper)]
-  Inspiratory wheeze rate for P2 breathing pattern [Insp Wheeze Rate(P2 : deeper)]
-  Inspiratory chest amplitude for P2 breathing pattern [Insp chest RMS score(P2 : deeper)]
-  Average lead and lag of chest channels compared to the tracheal channel for P2 breathing pattern [Lead score(P2 : deeper); Lag score(P2 : deeper)]
-  Inter-channel asynchrony at the beginning and end of inspiration for P2 breathing pattern [Lead STDev score (channel asynchrony independent of trachea sound) (P2 : deeper); Lag STDev score (channel asynchrony independent of trachea sound)(P2 : deeper)]
-  Lead time-integrated amplitude for P2 breathing pattern [Lead time-integrated amplitude(P2 : deeper)]
-  Lag time-integrated amplitude for P2 breathing pattern [Lag time-integrated amplitude(P2 : deeper)]
-  Ratio of low frequency energy to high frequency energy for P2 breathing pattern [Max R4 (low freq/high freq)(P2 : deeper)]
-  Ratio of duration of inspiration to the duration of expiration for P2 breathing pattern [R1 (Insp.Dur/Expir.Dur)(P2 : deeper)]
-  Ratios of peak inspiratory amplitude to peak expiratory amplitude for P2 breathing pattern [Ratio(peak insp amplitude/peak expir amplitude)(P2 : deeper)]
-  Dynamic range score(P2 : deeper)
-  Slope of the chest versus tracheal sound function during inspiration for P2 breathing pattern [Slope of chest vs trachea during Insp(P2 : deeper)]
-  Non-weighted total acoustic COPD scores for both breathing patterns [COPD total score NOTweighted(P1 : normal)], [COPD total score NOTweighted(P2 : deeper)]
-  Weighted total acoustic COPD scores for both breathing patterns [COPD total score weighted(P1 : normal)], [COPD total score weighted(P2 : deeper)]
The individual scores for each stethographics parameter (TEST) are presented as value (standard result, STRESN) for each subject, together with the visit number (VISITNUM) at which stethographics was performed. Click here for additional data file. Copyright: © 2018 Chaudhary N et al. 2018 Data associated with the article are available under the terms of the Creative Commons Zero "No rights reserved" data waiver (CC0 1.0 Public domain dedication).

Lung HRCT HRCT data for each subject are presented as scores for:
-  Bronchial wall thickening
-  Emphysema and Emphysema Type
-  Extent of Disease
-  Interstitial disease and Interstitial disease distribution
-  Interstitial Score
-  Nodules + other abnormality
-  Severity of bronchial dilatation
-  Small airways disease
-  Traction bronchiectasis
-  The sum of the severity of bronchial dilatation and traction bronchiectasis scores (Total Bronchiectasis Score)
-  The representative mean of the 5 component scores, i.e. Extent of Disease score, Severity of bronchial dilatation score, Traction bronchiectasis, Bronchial wall thickening score, and Small airways disease score (Total COPD CT Score)
The individual scores for each CT parameter (TEST) are presented as value (standard result, STRESN) together with the visit number (VISITNUM) at which HRCT scans were obtained. Click here for additional data file. Copyright: © 2018 Chaudhary N et al. 2018 Data associated with the article are available under the terms of the Creative Commons Zero "No rights reserved" data waiver (CC0 1.0 Public domain dedication).

Cardiopulmonary Exercise Tolerance Test Cardiopulmonary exercise tolerance test data, i.e. heart rate, gas exchange ratio or respiratory exchange ratio (RER), absolute and relative rates of maximum oxygen consumption (VO2; VO2/kg), absolute and relative percent predicted oxygen consumption (VO2 %Pred; VO2/kg %Pred), and carbon monoxide output (VCO2) for each subject. The data for each measurement (TEST) are presented as value (standard result, STRESN) and unit (standard unit, STRESU) for each subject, together with the visit number (VISITNUM) at which measurements were obtained. Click here for additional data file. Copyright: © 2018 Chaudhary N et al. 2018 Data associated with the article are available under the terms of the Creative Commons Zero "No rights reserved" data waiver (CC0 1.0 Public domain dedication).

Questionnaires Table listing individual scores for:
-  Modified Medical Research Council (MMRC) Dyspnoea Scale (MMRCDS for BODE)
-  Bodily Pain component of SF-36 questionnaire (SF-36_Bodily Pain)
-  General Health component of SF-36 questionnaire (SF-36_General Health)
-  Mental Health component of SF-36 questionnaire (SF-36_Mental Health)
-  Physical Functioning component of SF-36 questionnaire (SF-36_Physical Functioning)
-  Social Functioning component of SF-36 questionnaire (SF-36_Social Functioning)
-  Emotional Role Functioning component of SF-36 questionnaire (SF-36_Role Emotional)
-  Physical Role Functioning component of SF-36 questionnaire (SF-36_Role Physical)
-  Vitality component of SF-36 questionnaire (SF-36_Vitality)
-  Modified BODE index (mBODE Index)
The score for each assessment (TEST) is presented as value (standard result, STRESN) for each subject. Click here for additional data file. Copyright: © 2018 Chaudhary N et al. 2018 Data associated with the article are available under the terms of the Creative Commons Zero "No rights reserved" data waiver (CC0 1.0 Public domain dedication).

Basic Summary Statistics Summary statistics for dataset 2– dataset 8 were computed for each of the 4 study groups. When multiple measurements of a given endpoint were available for a given subject, the median of these measurements was used for the given subject. The dataset contains the dataset number where individual measurements come from (Dataset), the considered test (TEST) and corresponding unit (STRESU), the group used for computing summary statistics (Group), and the following summary statistics: sample size (N), average (Mean), and standard deviation (Std). Click here for additional data file. Copyright: © 2018 Chaudhary N et al. 2018 Data associated with the article are available under the terms of the Creative Commons Zero "No rights reserved" data waiver (CC0 1.0 Public domain dedication).

## Data availability

The data referenced by this article are under copyright with the following copyright statement: Copyright: © 2018 Chaudhary N et al.

Data associated with the article are available under the terms of the Creative Commons Zero "No rights reserved" data waiver (CC0 1.0 Public domain dedication). 

The main data are provided for the 240 subjects who completed the study (60 subjects per group). For some assessments, parameters were measured at 2 visits and the measurement for each visit is provided. Missing data were not replaced.

Clinical study data were transferred from the clinical site as locked SAS datasets, formatted according to the Study Data Tabulation Model or SDTM ( https://www.cdisc.org/standards/foundational/sdtm, accessed 16 May 2017). The data presented here was extracted from these standardized study datasets.

**Dataset 1. Demographics.** List of all unique study subject IDs (USUBJID) and demographic information including sex, age, COPD GOLD stage (if any), cigarette consumption (expressed as pack-years and cigarettes per day), study group (ARMCD), and the matching ID (MATCHID) allowing the pairing of control subjects to their ‘matched’ COPD subject.

DOI, 10.5256/f1000research.11698.d163781 ^25^

**Dataset 2. Vital Signs.** Table of body mass index, diastolic and systolic blood pressure, heart and respiration rate measures. The data for each measurement (TEST) are presented as value (standard result, STRESN) and unit (standard unit, STRESU) for each subject, and visit number (VISITNUM) at which measurements were obtained.

DOI, 10.5256/f1000research.11698.d163782 ^26^

**Dataset 3. Lung Function.** Table of lung function data for each subject, including percent predicted values for:

-   Forced expiratory volume in one second (FEV1 %Pred)
-   Forced vital capacity (FVC %Pred)
-   Ratio of FEV1 to maximum vital capacity (FEV 1 % VC MAX)
-   Functional residual volume by helium dilution technique (FRC-He %Pred)
-   Maximum expiratory flow at 25% of expired volume (MEF 25 %Pred)
-   Maximum expiratory flow at 75% of expired volume (MEF 75 %Pred)
-   Diffusion capacity (transfer factor) for carbon monoxide (TLCO %Pred)

The data for each measurement (TEST) are presented as value (standard result, STRESN), together with the visit number (VISITNUM) at which measurements were obtained.

DOI, 10.5256/f1000research.11698.d163783 ^27^

**Dataset 4. Impulse Oscillometry.** Table of impulse oscillometry data for each subject, including resistance at 5 (R at 5Hz) and 20 Hertz (R at 20Hz), reactance at 5 (X at 5Hz), and Resonant Frequency. The data for each measurement (TEST) are presented as value (standard result, STRESN), together with the visit number (VISITNUM) at which measurements were obtained.

DOI, 10.5256/f1000research.11698.d163784 ^28^

**Dataset 5. Stethographics.** Stethographics measures for each subject were obtained for 2 breathing patterns, also referred to as normal breathing pattern or P1 (P1 : normal) and deeper breathing or P2 (P2 : deeper). Data presented here include scores for:

-   Expiratory crackle rate for P2 breathing pattern [Expir Crackle Rate(P2 : deeper)]
-   Inspiratory crackle rate for P2 breathing pattern [Insp Crackle Rate(P2 : deeper)]
-   Expiratory wheeze rate for P2 breathing pattern [Expir Wheeze Rate(P2 : deeper)]
-   Inspiratory wheeze rate for P2 breathing pattern [Insp Wheeze Rate(P2 : deeper)]
-   Inspiratory chest amplitude for P2 breathing pattern [Insp chest RMS score(P2 : deeper)]
-   Average lead and lag of chest channels compared to the tracheal channel for P2 breathing pattern [Lead score(P2 : deeper); Lag score(P2 : deeper)]
-   Inter-channel asynchrony at the beginning and end of inspiration for P2 breathing pattern [Lead STDev score (channel asynchrony independent of trachea sound) (P2 : deeper); Lag STDev score (channel asynchrony independent of trachea sound)(P2 : deeper)]
-   Lead time-integrated amplitude for P2 breathing pattern [Lead time-integrated amplitude(P2 : deeper)]
-   Lag time-integrated amplitude for P2 breathing pattern [Lag time-integrated amplitude(P2 : deeper)]
-   Ratio of low frequency energy to high frequency energy for P2 breathing pattern [Max R4 (low freq/high freq)(P2 : deeper)]
-   Ratio of duration of inspiration to the duration of expiration for P2 breathing pattern [R1 (Insp.Dur/Expir.Dur)(P2 : deeper)]
-   Ratios of peak inspiratory amplitude to peak expiratory amplitude for P2 breathing pattern [Ratio(peak insp amplitude/peak expir amplitude)(P2 : deeper)]
-   Dynamic range score(P2 : deeper)
-   Slope of the chest versus tracheal sound function during inspiration for P2 breathing pattern [Slope of chest vs trachea during Insp(P2 : deeper)]
-   Non-weighted total acoustic COPD scores for both breathing patterns [COPD total score NOTweighted(P1 : normal)], [COPD total score NOTweighted(P2 : deeper)]
-   Weighted total acoustic COPD scores for both breathing patterns [COPD total score weighted(P1 : normal)], [COPD total score weighted(P2 : deeper)]

The individual scores for each stethographics parameter (TEST) are presented as value (standard result, STRESN) for each subject, together with the visit number (VISITNUM) at which stethographics was performed.

DOI, 10.5256/f1000research.11698.d163786 ^29^

**Dataset 6. Lung HRCT.** HRCT data for each subject are presented as scores for:

-   Bronchial wall thickening
-   Emphysema and Emphysema Type
-   Extent of Disease
-   Interstitial disease and Interstitial disease distribution
-   Interstitial Score
-   Nodules + other abnormality
-   Severity of bronchial dilatation
-   Small airways disease
-   Traction bronchiectasis
-   The sum of the severity of bronchial dilatation and traction bronchiectasis scores (Total Bronchiectasis Score)
-   The representative mean of the 5 component scores, i.e. Extent of Disease score, Severity of bronchial dilatation score, Traction bronchiectasis, Bronchial wall thickening score, and Small airways disease score (Total COPD CT Score)

The individual scores for each CT parameter (TEST) are presented as value (standard result, STRESN) together with the visit number (VISITNUM) at which HRCT scans were obtained.

DOI, 10.5256/f1000research.11698.d163788 ^30^

**Dataset 7. Cardiopulmonary Exercise Tolerance Test.** Cardiopulmonary exercise tolerance test data, i.e. heart rate, gas exchange ratio or respiratory exchange ratio (RER), absolute and relative rates of maximum oxygen consumption (VO2; VO2/kg), absolute and relative percent predicted oxygen consumption (VO2 %Pred; VO2/kg %Pred), and carbon monoxide output (VCO2) for each subject.

The data for each measurement (TEST) are presented as value (standard result, STRESN) and unit (standard unit, STRESU) for each subject, together with the visit number (VISITNUM) at which measurements were obtained.

DOI, 10.5256/f1000research.11698.d163789 ^31^

**Dataset 8. Questionnaires.** Table listing individual scores for:

-   Modified Medical Research Council (MMRC) Dyspnoea Scale (MMRCDS for BODE)
-   Bodily Pain component of SF-36 questionnaire (SF-36_Bodily Pain)
-   General Health component of SF-36 questionnaire (SF-36_General Health)
-   Mental Health component of SF-36 questionnaire (SF-36_Mental Health)
-   Physical Functioning component of SF-36 questionnaire (SF-36_Physical Functioning)
-   Social Functioning component of SF-36 questionnaire (SF-36_Social Functioning)
-   Emotional Role Functioning component of SF-36 questionnaire (SF-36_Role Emotional)
-   Physical Role Functioning component of SF-36 questionnaire (SF-36_Role Physical)
-   Vitality component of SF-36 questionnaire (SF-36_Vitality)
-   Modified BODE index (mBODE Index)

The score for each assessment (TEST) is presented as value (standard result, STRESN) for each subject.

DOI, 10.5256/f1000research.11698.d163790 ^32^

**Dataset 9. Basic Summary Statistics.** Summary statistics for dataset 2– dataset 8 were computed for each of the 4 study groups. When multiple measurements of a given endpoint were available for a given subject, the median of these measurements was used for the given subject. The dataset contains the dataset number where individual measurements come from (Dataset), the considered test (TEST) and corresponding unit (STRESU), the group used for computing summary statistics (Group), and the following summary statistics: sample size (N), average (Mean), and standard deviation (Std).

DOI, 10.5256/f1000research.11698.d196240 ^33^

## Consent

The study protocol number QASMC202 was reviewed and approved by The Black Country Ethics Committee, a UK National Health Service (NHS) Ethics Committee (reference number 11/WM/0114).

Written informed consent to collect and use personal data such as age, gender, ethnicity and medical/surgical history, and to publish these results was obtained from all study participants. None of the study participants can be identified by the sponsor by using any of the data provided in this data note.

## Supplementary material

**Supplementary Material S1: Inclusion and exclusion criteria.**

**Supplementary Material S2. Removal/withdrawal of subjects from the study.**

**Supplementary Material S3. Study restrictions.**

**Supplementary Material S4. Schedule of events.**
