# Supplementary material for: Physiological and biological characterization of smokers with and without COPD
Source: F1000Res. 2018 Mar 7;6:877. Originally published 2017 Jun 13. [Version 2] doi: 10.12688/f1000research.11698.2 (PMC5843826; doi:10.12688/f1000research.11698.2)
Supplement: Supplementary file 10 [file f1000research-6-15389-s0009.tgz › ed8e41a3-60ac-4a2b-903d-a8707cc7e80f.pdf]

**Supplementary Material S1: Inclusion and exclusion criteria**

Subjects were to fulfil the following criteria for inclusion in the study:

1. Provision of written, signed informed consent including consent for genetic testing.
2. Ability to comply with study procedures.
3. Males and females aged 40-70 years inclusive.
4. Body mass index (BMI) of 18 to 35 kg/m2 inclusive and a minimum body weight of 50 kg.
5. Normal physical examination and normal laboratory values, 12-lead ECG and vital signs (BP, heart rate [HR] and respiratory rate), unless the investigator considered an abnormality to not be clinically significant.
6. Ability to perform reproducible spirometry according to the American Thoracic Society and the European Respiratory Society (ATS/ERS) guidelines (Miller, 2005).
7. Ability to produce a minimum 0.1 g sputum sample after induction with inhaled hypertonic saline.

Additional inclusion criteria for the COPD group were:

1. A clinical diagnosis of COPD according to the GOLD guidelines (stage 1-2).
2. Current smokers with ≥10 pack-year smoking history.
3. Demonstrate a post-bronchodilator $FEV_1$/FVC ratio of <70% and $FEV_1$ of ≥50% of the predicted normal.

Additional inclusion criteria for the never-smoker group were:

1. Never smoked tobacco products.
2. Demonstrated normal lung function by post-bronchodilator $FEV_1$ of ≥80% of the predicted normal, with no evidence of airway obstruction and an $FEV_1$/FVC ratio of ≥70%.
3. Sputum eosinophilia of <2% based on the sample collected at visit 1.
4. Total leukocyte count per gram sputum of <20 $x10^6$ cells, to exclude subjects with sub-clinical infection who did not present with clinical symptoms.

Additional inclusion criteria for the smoker group were:

1. Current smoker with a defined smoking history of ≥10 pack years.
2. Demonstrated normal lung function by post-bronchodilator $FEV_1$ of ≥80% of the predicted normal, with no evidence of airway obstruction and an $FEV_1$/FVC ratio of ≥70%.

Additional inclusion criteria for the former smoker group were:

1. Ex-smoker with a defined smoking history of ≥10 pack years and having quit smoking at least 1 year before entering the study.
2. Demonstrated normal lung function by post-bronchodilator FEV1 of ≥80% of the predicted normal, with no evidence of airway obstruction and an FEV1/FVC ratio of ≥70%.

Subjects who met any of the following criteria were excluded from the study:

1. Current evidence or recent history of any clinically significant disease or abnormality (other than COPD for subjects in the COPD group), which in the opinion of the investigator would have put the subject at risk or compromise the quality of the study data (including but not limited, to cardiovascular disease, myocardial infarction, cardiac failure, uncontrolled hypertension, life-threatening arrhythmia, uncontrolled diabetes, neurologic or neuromuscular disease, liver disease, gastrointestinal disease or electrolyte abnormalities).
2. Females with a positive pregnancy test at visit 1 or 3.
3. Females currently breastfeeding.
4. Involvement in the planning and conduct of the study.
5. Surgery or significant trauma within 3 months of visit 1.
6. History of tuberculosis or other non-specific pulmonary disease such as asthma.
7. Symptoms, signs or laboratory findings suggestive of an ongoing infectious illness as judged by the investigator at visit 1 or 2.
8. Participation in any clinical study with an investigational drug in the 4 months prior to visit 1, participation in a study with a new formulation of a marketed drug in the 3 months prior to visit 1, or participation in a methodology study in the month prior to visit 1.
9. Symptoms of any clinically significant illness within the 2 weeks prior to visit 1.
10. Significant history of alcohol abuse or consumption of more than the recommended units of alcohol per week (28 units for males and 21 units for females).
11. Significant history of drug abuse (including benzodiazepines) or a positive drugs of abuse test at visit 1.
12. Subjects, who in the opinion of the investigator should not, for reasons of safety or compliance, participate in the study.
13. Use of prohibited medications, as specified in S3.
14. Subjects with a first-degree relative (parent, sibling or child) already enrolled in the study.

Additional exclusion criteria for the COPD group were:

1. Recent history of hospitalization due to exacerbation of airway disease within 3 months or a need for increased COPD treatment within the 6 weeks prior to the screening visit.
2. Prior lung volume reduction surgery or a history of chest/lung irradiation.
3. Regular use of daily oxygen therapy.
4. A long-standing history and primary diagnosis of asthma.
5. Use of systemic steroids within the 3 months prior to the screening visit.
6. Respiratory tract infection within the 6 weeks prior to the screening visit.
