# Supplementary material for: Physiological and biological characterization of smokers with and without COPD
Source: F1000Res. 2018 Mar 7;6:877. Originally published 2017 Jun 13. [Version 2] doi: 10.12688/f1000research.11698.2 (PMC5843826; doi:10.12688/f1000research.11698.2)
Supplement: Supplementary file 11 [file f1000research-6-15389-s0010.tgz › 121130a6-691c-47ad-9440-c0bcca16df4e.pdf]

Supplementary Material S2. Removal/withdrawal of subjects from the study

Each subject was informed of his or her right to withdraw from the study at any time and for any reason. The investigator could withdraw a subject from the study at any time if they considered that the subject's health might be compromised by remaining in the study or if the subject was not sufficiently cooperative.

Subjects could be withdrawn from the study for any of the following reasons:

- Experiencing a serious or intolerable adverse event
- Requiring a medication prohibited by the protocol
- Pregnancy
- Non-compliance with study restrictions or unable to commit to the study visits
- Lost to follow up
- Withdrawal of informed consent
- Current smokers with normal lung function (post-bronchodilator FEV_1 of ≥80% of the predicted normal, with no evidence of airway obstruction and an FEV_1/FVC ratio of ≥70% at visit 1) and abnormal transfer factor (TLCO of <60%) at visit 2 were withdrawn at visit 3
- Former smokers with normal lung function (post-bronchodilator FEV_1 of ≥80% of the predicted normal, with no evidence of airway obstruction and an FEV_1/FVC ratio of ≥70%) and abnormal transfer factor (TLCO of <60 %) at visit 2 were withdrawn at visit 2

Subjects could also be withdrawn at any time or for any reason if the investigator concluded that it was in their best interest. Protocol violations did not lead to subject withdrawal, unless they represented a significant risk to the subject's safety. Subjects could voluntarily withdraw from the study for any reason at any time. Subjects were considered withdrawn if they withdrew consent, failed to return for study visits, or became lost to follow up for any other reason.

Prior to discontinuing a subject from the study, the investigator contacted the sponsor or designee and the reason for withdrawal or termination was recorded.

If a subject prematurely discontinued the study for any reason, every effort was made to complete the follow-up call. If a subject withdrew from the study with a clinically significant laboratory abnormality (at screening) or an adverse event, every effort was made to follow up these events to satisfactory resolution or until the investigator considered the event stable or chronic in nature.

For subjects who were lost to follow up (e.g., subjects whose status was unclear because they were not contactable for the follow-up telephone call, but who had not stated an intention to withdraw), the investigator was to show due diligence by documenting the steps taken to contact the subject.
