# Supplementary material for: Physiological and biological characterization of smokers with and without COPD
Source: F1000Res. 2018 Mar 7;6:877. Originally published 2017 Jun 13. [Version 2] doi: 10.12688/f1000research.11698.2 (PMC5843826; doi:10.12688/f1000research.11698.2)
Supplement: Supplementary file 12 [file f1000research-6-15389-s0011.tgz › d74dd83e-472c-44c0-a710-e3760ffc8b10.pdf]

Supplementary Material S3. Study restrictions

1. Permitted medications, medication withholding and study restrictions

The use of the following medications was permitted between study visits, provided that they were maintained at a stable dose during the study and were withheld prior to visit 1 (screening 1), visit 2, and visit 4 and for the durations specified:

- Theophylline for at least 24 hours
- Long-acting β2-agonists (e.g., formoterol, salmeterol) for at least 24 hours
- Short-acting β2-agonists (e.g., salbutamol) for at least 8 hours. Rescue salbutamol was allowed for as-needed use at any time during the study, however, if rescue salbutamol was used within 8 hours of a study visit the visit was to be rescheduled.
- Short-acting anticholinergics (e.g., ipratropium) for at least 8 hours
- Combination short-acting bronchodilators (e.g., salbutamol/ipratropium) for at least 8 hours
- Long-acting anticholinergics (e.g., tiotropium) for at least 48 hours
- Inhaled steroids (e.g., fluticasone, budesonide) for at least 48 hours
- Combinations of inhaled steroids and long-acting β2-agonists (e.g., fluticasone/salmeterol, budesonide/formoterol) for at least 48 hours prior to study visits

These medications could be administered after lung function procedures at all visits.

2. Permitted medications

The following medications could be taken during the course of the study. Any medications not listed were subject to approval by the investigator:

- Inhaled steroids (if the dose had been stabilized for at least 6 weeks prior to the screening visit and was stable throughout the study period)
- Topical or ophthalmic corticosteroids
- Contraceptives

3. Prohibited medications

Use of the following medications was prohibited during the course of the study:

- Systemic corticosteroids (for 3 months prior to and for the duration of the study)
- Intranasal corticosteroids (for 3 months prior to and for the duration of the study)

4. Study restrictions

Subjects were required to:

- Abstain from consuming products containing poppy seeds for the 7 days prior to visit 1
- Abstain from drinking alcohol for the 48 hours prior to all visits
- Abstain from strenuous physical activity (defined as anything different from the subject’s normal physical routine) for the 3 days prior to all visits
- Attend all study visits after a light breakfast
- Abstain from intake of any food or drink containing caffeine for the 8 hours prior to all lung function testing

- Abstain from eating foods rich in nitrates for the 24 hours prior to visits 2 and 4
- Abstain from blood donation during the study, unless as part of study procedures
- Not use any prohibited medication
- Females were to avoid becoming pregnant for the duration of the study
