# Supplementary material for: Physiological and biological characterization of smokers with and without COPD
Source: F1000Res. 2018 Mar 7;6:877. Originally published 2017 Jun 13. [Version 2] doi: 10.12688/f1000research.11698.2 (PMC5843826; doi:10.12688/f1000research.11698.2)
Supplement: Supplementary file 13 [file f1000research-6-15389-s0012.tgz › 18824efb-8628-4e32-9b57-86a0d36fdea6.pdf]

Supplementary Material S4. Schedule of events.

**Assessment**	**Visit 1** (Screening)	**Visit 1a**[1] (1-21 days after visit 1)	**Visit 1b**[2] (within 28 days of visit 2)	**Visit 2** (4-28 days after visit 1)	**Visit 3** (3-14 days after visit 2)	**Visit 4** (3-14 days after visit 3)	**Follow-up call** (3-10 days after visit 4)
Informed consent (with genetic consent)	✓[3]						
Smoking cessation information	✓[4]						✓[4]
Inclusion/exclusion criteria	✓	✓	✓	✓	✓	✓	
Demographics	✓						
Height and weight	✓		✓				
Medical/surgical history	✓	✓	✓				
Physical examination	✓		✓		✓		
Lung CT scan					✓		
12-lead ECG	✓		✓		✓[5]		
Continuous cardiac monitoring					✓		
Vital signs	✓		✓	✓	✓[5]	✓	
Alcohol breath test	✓		✓				
Plasma cotinine test	✓[6]		✓[6]				
Drugs of abuse test	✓		✓				
Clinical chemistry & haematology	✓		✓				
Serum pregnancy test	✓[7]		✓[7]				
Urinalysis	✓		✓				
Urine pregnancy test					✓[8]		
Salbutamol administration	✓[9, 10]	✓[10]	✓[9]	✓[10]	✓[10]	✓[10]	
Full lung function test	✓[11]		✓	✓[12]		✓	
Cardio pulmonary exercise test ($VO_{2\,max}$ & SpO_2)					✓[13]		
Differential exhaled FENO				✓		✓	
Impulse oscillometry				✓		✓	
Computerized multichannel lung sounds analysis (stethographics)				✓		✓	
Sputum induction	✓[14,15]	✓		✓	✓[16]	✓	

Assessment	Visit 1 (Screening)	Visit 1a[1] (1-21 days after visit 1)	Visit 1b[2] (within 28 days of visit 2)	Visit 2 (4-28 days after visit 1)	Visit 3 (3-14 days after visit 2)	Visit 4 (3-14 days after visit 3)	Follow-up call (3-10 days after visit 4)
Exhaled breath temperature				✓		✓	
Nasal sampling				✓[17]		✓[17]	
Blood sample for DNA and proteomics				✓			
Blood biomarker sample				✓			
Lipidomics blood sample				✓			
QOL questionnaires and MMRC Dyspnoea Scale				✓[18]			
Adverse events	✓	✓	✓	✓	✓	✓	✓
Concomitant medication	✓	✓	✓	✓	✓	✓	✓

Key:

1. Subjects who failed to produce an adequate sputum sample at screening could (at the discretion of the investigator or suitably qualified designee) be invited to attend an additional visit to produce an adequate sample.
2. Never-smokers who had produced sputum and been classed as screening failures due to a high neutrophil count (a neutrophil count <80% of the total number of leukocytes per gram of sputum) were invited to the clinic for a repeat screening visit (visit 1b) without sputum induction. If the subject met the inclusion/exclusion criteria they were enrolled into the study. The sputum sample produced at the original screening visit was used for analysis.
3. Informed consent could be obtained at the registration/review visit conducted prior to visit 1.
4. Current smokers only
5. ECG and vital signs were taken before and 10 minutes after cardio-pulmonary exercise testing; vital signs were assessed at intermittent intervals during cardio-pulmonary exercise testing.
6. Plasma cotinine was tested for all subjects. Subjects in the never- and former smoker groups with a positive plasma cotinine test were withdrawn from the study.
7. Performed for female subjects of childbearing potential only.
8. Performed for all female subjects of childbearing potential prior to CT scan.
9. 400 µg salbutamol from a metered dose inhaler was administered via a spacer at visit 1, 15-20 minutes before repeat measurement of FEV_1 and FVC.
10. 200 µg salbutamol MDI was administered via a spacer to subjects with an FEV_1 of ≤80% 15 minutes before sputum induction.
11. FEV_1 and FVC only. Screening spirometry measurements were repeated post-bronchodilator.
12. The following withdrawal process applied:
    - Current smokers with normal lung function (post-bronchodilator FEV_1 of ≥80% of the predicted normal with no evidence of airway obstruction and an FEV_1/FVC ratio of ≥70% at visit 1) and abnormal transfer factor (TLCO <60% at visit 2) were withdrawn at visit 3. A follow-up call was to be made 3 to 10 days after the last visit.
    - Former smokers with normal lung function (post-bronchodilator FEV_1 of ≥80% of the predicted normal, with no evidence of airway obstruction and an FEV_1/FVC ratio of ≥70% at visit 1) and abnormal transfer factor (TLCO <60% at visit 2) were withdrawn at visit 2. A follow-up call was to be made 3 to 10 days after the last visit.

13. Cardio-pulmonary exercise tests (VO_{2max} and SpO_2) were not performed for current smokers with normal lung function (post bronchodilator FEV_1 of ≥80% of the predicted normal, with no evidence of airway obstruction and an FEV_1/FVC ratio of ≥70% at visit 1) and abnormal transfer factor (TLC0 <60% at visit 2) at visit 3.
14. Subjects also underwent sputum induction assessment to test whether they could produce a sputum sample of ≥0.1 g.
15. Never-smokers who had previously produced sputum and were screening failures due to a high neutrophil count (a neutrophil count of >80% of the total number of leukocytes per gram sputum), were invited to the clinic for repeat screening (visit 1b) without sputum induction.
16. An additional sputum induction procedure was only conducted if the subject did not produce an adequate sputum sample at visit 2.
17. Nasal sampling to include nasal lavage and nasal scrape.
18. SF-36, a general health questionnaire, and MMRC Dyspnoea Scale questionnaire.
