# Supplementary material for: Physiological and biological characterization of smokers with and without COPD
Source: F1000Res. 2018 Mar 7;6:877. Originally published 2017 Jun 13. [Version 2] doi: 10.12688/f1000research.11698.2 (PMC5843826; doi:10.12688/f1000research.11698.2)
Supplement: Peer review history

# Peer review: Referee response for version 2

**DOI:** 10.5256/f1000research.15389.r31598

**Published:** 2018-03-07

**Authors:** Amany F. Elbehairy

**Affiliations:** 1 Department of Medicine, Queen’s University, Kingston, ON, Canada

Thanks for the authors for addressing my concerns. I have no further comments.

I have read this submission. I believe that I have an appropriate level of expertise to confirm that it is of an acceptable scientific standard.

# Peer review: Referee response for version 1

**DOI:** 10.5256/f1000research.12639.r25445

**Published:** 2017-09-14

**Authors:** David Mannino

**Affiliations:** 1 Department of Preventive Medicine and Environmental Health, University of Kentucky School of Medicine, Lexington, KY, USA

The authors provide data from a Phillip Morris sponsored study that looks at biomarkers of lung health in a groups of people with COPD and "healthy" current, former and never smokers.  The data is provided- but no analysis is done of the raw data.

 I believe summary tables of the key features of the database would be a useful addition.

I have read this submission. I believe that I have an appropriate level of expertise to confirm that it is of an acceptable scientific standard.

# Response

**Published:** 2018-02-28

**Authors:** Gregory Vuillaume

**Affiliations:** Philip Morris, Switzerland

We appreciate the positive feedback. While our intention was to provide the data describing the physiological characteristics of the study population, we understand the reviewer’s point of view and prepared a table of summary statistics for all of the reported endpoints.

# Peer review: Referee response for version 1

**DOI:** 10.5256/f1000research.12639.r23964

**Published:** 2017-07-20

**Authors:** Amany F. Elbehairy

**Affiliations:** 1 Department of Medicine, Queen’s University, Kingston, ON, Canada

The authors presented detailed data across groups of smokers with and without COPD and healthy non-smokers. Data are well presented and methodology is detailed. I have 3 comments/suggestions.

1. The title is very long and need to be more concise. Suggestions:  "Physiological and biological characterization of smokers with and without COPD" .
2. The authors used FEV1/FVC fixed ratio of 0.7 to diagnose COPD among their smokers. Would the data be different if they used both criteria of having FEV1/FVC less than 0.7 and less than the lower limit of normal to diagnose COPD?
3. Data presented for CPET are the peak values. Data at standardized work rate or ventilation can give better characterization of in-between groups differences.

I have read this submission. I believe that I have an appropriate level of expertise to confirm that it is of an acceptable scientific standard, however I have significant reservations, as outlined above.

# Response

**Published:** 2018-02-28

**Authors:** Gregory Vuillaume

**Affiliations:** Philip Morris, Switzerland

1. We are grateful for the reviewer’s feedback and followed the advice to shorten the title accordingly.
2. We are uncertain whether we understand this comment correctly. The reference population of never-smokers in this study was rather small (N=60), and there was considerable variation in the lung function data. Considering the lower limit of normal, i.e. the 5 ^th^ percentile of the FEV1/FVC observed in never-smokers, in addition to the 2009 GOLD criterion of FEV1/FVC <0.7, we believe we would not have misclassified the current smokers as being “healthy”. In brief, therefore, we do not think that the data would have been different.
3. This is valuable feedback, which we appreciate. Unfortunately, we only have the peak values for the CPET and cannot study potential between-group differences further.
